# Vitamin-B12 status among the mother–infant dyad: an explorative overview in a single centre in Lombardy

**DOI:** 10.3389/fnut.2026.1903144

**Published:** 2026-09-11

**Authors:** Francesca Furlan, Francesco Tagliaferri, Stefano Mariani, Cristiana Berti, Simona Salera, Simona Lucchi, Simona Ferraro, Cristina Cereda, Giulia Fiore, Martina Tosi, Alice Spano, Francesco Galandrini, Carlo Agostoni, Elvira Verduci, Francesca Menni

**Affiliations:** 1 Fondazione IRCCS Ca’ Granda Ospedale Maggiore Policlinico, Pediatric Unit, Milan, Italy; 2 Department of Clinical Sciences and Community Health, Dipartimento di Eccellenza, University of Milan, Milan, Italy; 3 Department of Neuroscience, Rehabilitation, Ophthalmology, Genetics, Maternal and Child Health, University of Genoa, Genoa, Italy; 4 Center of Functional Genomics and Rare Diseases, Department of Pediatrics, Buzzi Children's Hospital, Milan, Italy; 5 Department of Biomedical and Clinical Sciences (DIBIC), University of Milan, Milan, Italy; 6 Department of Health Sciences, University of Milan, Milan, Italy; 7 Postgraduate School of Paediatrics, University of Milan, Milan, Italy

**Keywords:** expanded newborn screening, homocysteine, methylmalonic acid, pregnancy, vegetarian, vitamin B 12 deficiency

## Abstract

**Introduction:**

Maternal vitamin B12 deficiency is an increasingly recognized cause of secondary neonatal cobalamin deficiency and can be incidentally detected through expanded newborn screening.

**Methods:**

We retrospectively evaluated 240 infants and 238 mothers referred to a Regional Clinical Centre in Lombardy, Italy, between 2016 and 2026 following a positive newborn screening result suggestive of methylmalonic acidemia or vitamin B12 deficiency. Clinical, dietary, and biochemical data were collected at diagnosis and during follow-up after vitamin B12 supplementation and complementary feeding introduction.

**Results:**

At baseline, 56.2% of infants had vitamin B12 concentrations below the laboratory reference range, while elevated homocysteine and plasma methylmalonic acid were observed in 52.8 and 44.8% of cases, respectively. Maternal and neonatal vitamin B12 concentrations were positively correlated, and infants born to vitamin B12-deficient mothers showed significantly lower vitamin B12 levels than those born to mothers with adequate status. Vegetarian mothers had lower vitamin B12 concentrations than omnivorous mothers, whereas exclusively breastfed infants showed lower vitamin B12 concentrations than formula-fed infants. Compared with conventional laboratory reference ranges, the National Institute for Health and Care Excellence criteria identified significantly more infants with biochemical evidence of functional vitamin B12 deficiency. Following supplementation, vitamin B12 concentrations increased markedly and functional biomarkers normalized in almost all infants, with improvements maintained after supplementation discontinuation.

**Discussion:**

These findings confirm the strong influence of maternal vitamin B12 status on neonatal biochemical status, support the role of newborn screening in identifying secondary neonatal vitamin B12 deficiency, and suggest that currently adopted laboratory reference intervals may underestimate the burden of clinically relevant vitamin B12 deficiency in mother–infant dyads.

## 1 Introduction

Vitamin B12 (B12), also known as cobalamin (Cbl) is an essential nutrient in human that obtain it by foods of animal origin. B12 plays an important role in the normal metabolism and function of a number of organ systems (1, 2). Vitamin B12 is a cofactor of methionine synthase and methylmalonyl-CoA mutase, so its deficiency leads to high concentrations of homocysteine (Hcy) and methylmalonic acid (MMA). Several biomarkers exist to evaluate B12 status. Total serum/plasma cobalamin is the most widely used biomarker of vitamin B12 status, and the National Institute for Health and Care Excellence (NICE) has recently proposed diagnostic cut-offs for its interpretation (3). Serum holotranscobalamin, plasma Hcy, and serum/urine MMA are sensitive functional markers of vitamin B12 deficiency, but their use is limited by higher costs and lower availability in routine clinical practice (2, 4, 5). A reduction in serum B12 concentration is indicative indeed of prolonged deficiency, and may not manifest for several years, while an increase in MMA and Hcy occurs earlier (6).

Macrocytic and megaloblastic anaemia, myelopathy, neuropathy, and neuro-psychiatric disturbances are the main clinical manifestations of B12 deficiency (7). Low levels could arise, not only from an inadequate intake as a result of the diet, but also from malabsorption and genetic defects altering transport (2). The risk of B12 insufficiency is strong for pregnant and lactating women, and for infants and young children as well (2). In this regard, the EURopean micronutrient RECommendations Aligned Network of Excellence, prioritized vitamin B12 among the micronutrients of public health relevance within these populations (8). There is evidence suggesting that pregnant women with a low folate supply combined with low blood B12 levels have a drastically increased risk of neural tube defects (9). Some ‘placenta events’ likely arise from deficiencies of either folate and/or Cbl or defects within the methionine–homocysteine metabolic pathways (10). Elevated tHcy levels are associated with an increased risk of low birth weight (LBW) (2).

Breast milk vitamin B12 concentrations are related to maternal status in pregnancy and postpartum, to point-out that a poor maternal status may affect the foetus and therefore the infant (11). Newborns from B12 deficient mothers have compromised liver stores and consume milk with a lower vitamin level, being thereby at an increased risk for B12 deficiency and neurologic/developmental consequences (12). At the same time, the impact of maternal dietary intake of vitamin B12 must not be overlooked. B12 is commonly present in animal foods, for example, fish, dairy products and eggs; limiting its availability in exclusively plant-based diets (13). In this regard, the increasing adoption of vegetarian and vegan dietary pattern during pregnancy and infancy has gained particular attention (14).

Literature emphasizes a number of knowledge gaps in the field of B12 among childbearing-age and paediatric populations, deriving from the paucity of high-quality studies, and the biomarkers/reference intervals hampering B12 insufficiency diagnostic-specificity as well (2, 5, 15). In Italy, previous studies have assessed vitamin B12 status and related biochemical markers in mother–infant dyads identified through neonatal screening, with the aim of exploring the related neonatal vitamin B12 deficiency (14, 16, 17).

In light of the available literature, this exploratory study investigated vitamin B12 status in mother–infant dyads identified through incidental findings during the routine expanded newborn screening (NBS) Program at a tertiary referral center in Lombardy. The primary objective was to describe the profile of B12 biomarkers of both mothers and infants and to identify maternal determinants potentially contributing to neonatal vitamin B12 deficiency. A secondary objective was to assess changes in vitamin B12 status over time after clinical intervention, monitoring the mother–infant dyad up to 8 months of age.

## 2 Materials and methods

In Italy, from 2016 screening of about 40 metabolic diseases in newborns has been made mandatory (LEGGE 19 Agosto 2016, n. 167). Expanded NBS helps to identify many severe disorders in pre-symptomatic phase, simply by investigating by Tandem Mass Spectrometry (TMS) newborns’ Dried Blood Spots (DBS), taken in the first days of life (48–72 h) (7, 18). This panel includes organic acidemias, in particular Methylmalonic acidaemia and Cbl intracellular defects that are identified through the increase of propionyl carnitine (C3). For differential diagnosis a second-tier test (2TT) is then performed to test levels of Hcy, methionine (Met) and MMA on DBS.

The present study was conducted at the Paediatric Unit of Fondazione IRCCS Ca′ Granda Ospedale Maggiore Policlinico (Milan, Italy), one of the four Regional Clinical Centres (RCC) responsible for the confirmatory assessment and clinical management of newborns identified through the Lombardy Expanded NBS Program. Available baseline and follow-up clinical and biochemical data from newborn with positive expanded NBS results for Organic Acidemia/Vitamin-B12 Deficiency herein collected were analysed retrospectively.

### 2.1 Population and screening

We have retrospectively analysed all patients recalled to our RCC between July 2016 and February 2026 with an Organic Acidemia/Vitamin B12 deficiency risk condition. Of all mothers tested positive to NBS, only those who had been diagnosed with vitamin B12 deficiency were included in the retrospective study.

All newborns recalled underwent routine examinations, comprehensive of biochemical assessments and metabolic specific analysis (plasmatic acylcarnitines, and organic acids and only MMA on urine sample). The molecular analysis, to find mutations in the genes involved in the Cbl intracellular metabolism, was performed only when the values of plasmatic/urinary MMA or Hcy resulted persistently elevated despite modestly reduced B12 values. Depending on the biochemical values found, a different genetic panel was investigated for the search for mutations in genes responsible for the transport, distribution and intracellular metabolism of cobalamin.

### 2.2 Management and follow up

**Figure 1:**
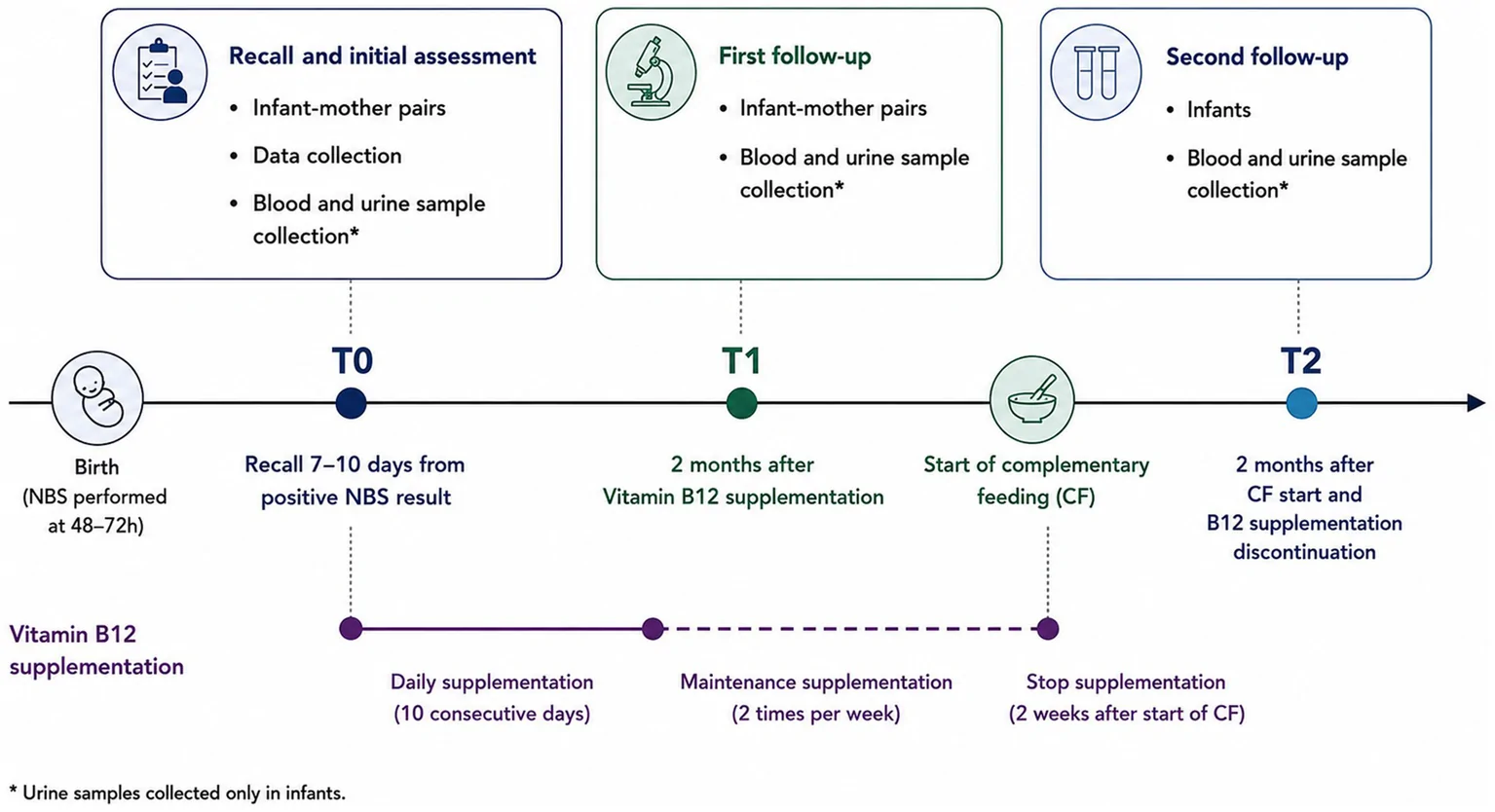
Study timeline and management pathway for newborns recalled after a positive newborn screening result suggestive of vitamin B12 deficiency. This figure was developed with the assistance of ChatGPT 5.5 through an iterative process which is described in Supplementary File 1.

Figure 1 presents patients management and follow up during the study, including clinical evaluation, biochemical examinations and Vitamin-B12 supplementation. Briefly:

- T0 [baseline]: after recall by the Regional Reference Laboratory for the Neonatal Screening (RRLNS); on indication of our reference centre medical records, dietary information and laboratory parameters were collected for the infants at the birthplace. Afterwards, vitamin-B12 supplementation was prescribed as explained below;
- T1 [after 2-months vitamin-B12 supplementation]: biomarkers were assessed to evaluate the responsiveness to vitamin-B12 supplementation;
- T2 [after 2-months of complementary feeding and stop of B12 supplementation]: biochemical parameters were gathered to evaluate the effect of stop B12 and introduction of animal foods on B12 status biomarkers.

At each point we assessed plasma ammonium, blood count parameters, hepatic function, homocysteine, plasma amino acids, plasmatic and urinary MMA. Contemporaneously at T0, only biochemical tests were prescribed to mothers. Blood and urine samples were analysed according to the hospital procedures and methods.

Additional investigations to identify the underlying cause of maternal vitamin B12 deficiency were not systematically performed throughout the study period. Due to changes in clinical practice between 2017 and 2026, tests for vitamin B12 malabsorption (including celiac disease, *Helicobacter pylori* infection, parasitosis, and atrophic gastritis) were initially reserved for mothers with an inadequate response to supplementation and were subsequently extended to all mothers undergoing evaluation.

### 2.3 Vitamin B12 dose and timing

Vitamin B12 supplementation with hydroxocobalamin (Neocytamen®) was initiated at baseline (T0). Infants received 1 mg/day orally for 10 days, followed by 2 mg/week until the introduction of complementary feeding (including animal-source foods). Mothers received the same regimen throughout the breastfeeding period. Since late 2017, infants have additionally received a single 1 mg intramuscular dose at the baseline visit. Vegetarian and vegan mothers were advised to continue vitamin B12 supplementation long-term.

### 2.4 Clinical parameters and dietary aspects

Demographic, perinatal, and clinical data were retrospectively collected. Infant variables included sex, parental consanguinity, twinning, mode of delivery, birth weight and percentile, gestational age, and feeding type (breastfeeding and formula feeding). Maternal variables included age at delivery and ethnicity.

During the clinical evaluation, all mothers were interviewed regarding their dietary pattern and the consumption frequency of animal-source foods (meat, fish, eggs, and dairy products). Dietary information was self-reported during routine clinical assessment and recorded in the clinical charts. Based on this information recalled in clinical records, mothers were classified into two main dietary groups: omnivorous (OM) (including flexitarian diets, defined as predominantly plant-based diets with occasional consumption of meat and meat products) and vegetarian (VEG) (including pescatarian, lacto-ovo vegetarian, ovo-vegetarian, and vegan diets). Owing to the retrospective nature of the study and the limited level of detail consistently available in the clinical records, further subclassification of the different vegetarian dietary patterns was not feasible.

### 2.5 Biochemical parameters

All data analysed were collected retrospectively. The following biochemical parameters performed at T0 were collected for newborns: plasma B12 (pg/mL), plasma MMA (μmol/L), urinary MMA (mmol/mol creatinine), plasma Hcy (μmol/L), and mean corpuscular volume (MCV; fl). In mothers, the T0 evaluation included plasma B12 (pg/mL), plasma Hcy (μmol/L), and MCV (fl), as well as plasma folate (ng/mL), haemoglobin (Hb; g/dL), and haematocrit (Ht; %) to assess haematological status potentially associated with chronic vitamin B12 deficiency.

Plasma vitamin B12 concentrations at baseline were measured for both mother and infants in the reference laboratory of our centre using electrochemiluminescence immunoassay (ECLIA), with a laboratory reference range of 191–663 pg/mL. Follow-up analyses were performed at our centre for infants, whereas mothers underwent laboratory testing at external facilities according to routine adult care pathways. Consequently, B12 status was further classified according to the National Institute for Health and Care Excellence (NICE) criteria as follows: serum vitamin B12 concentrations <180 pg/mL (pg/mL is equal to ng/L from the original reference) were considered indicative of confirmed deficiency, values between 180 and 350 pg/mL of probable deficiency, and values >350 pg/mL of no deficiency.

### 2.6 Statistical analysis

For descriptive statistics, qualitative data are reported as frequency (%). Quantitative data are presented as median. When only values below the laboratory detection limit were available (e.g., plasma B12 reported as <100 pg/mL), the corresponding threshold value was conventionally assigned for statistical analyses (e.g., 100 pg/mL). Continuous variables were compared between groups using the non-parametric Mann–Whitney *U* test (2 groups) or Kruskal–Wallis test with Dunn’s *post-hoc* test (in case of more than 2 groups). The Wilcoxon signed-rank test was used to evaluate two-paired groups. Spearman’s rank correlation coefficient (Spearman’s *ρ*) was performed to assess the relationship between two continuous variables. Lastly, the McNemar test was used to compare the proportion of false-negative obtained using the laboratory B12 cut-off and the NICE criteria, considering elevated plasma Hcy and MMA as the reference indicator of functional vitamin B12 deficiency. Statistical tests were two-sided; a *p* value less than 0.05 was considered statistically significant. Data were analysed using SPSS software (version 25). Box-and-whisker plots were generated using GraphPad Prism version 8 (GraphPad Software, San Diego, CA, USA), displaying median and interquartile range for each time point.

**Figure 2:**
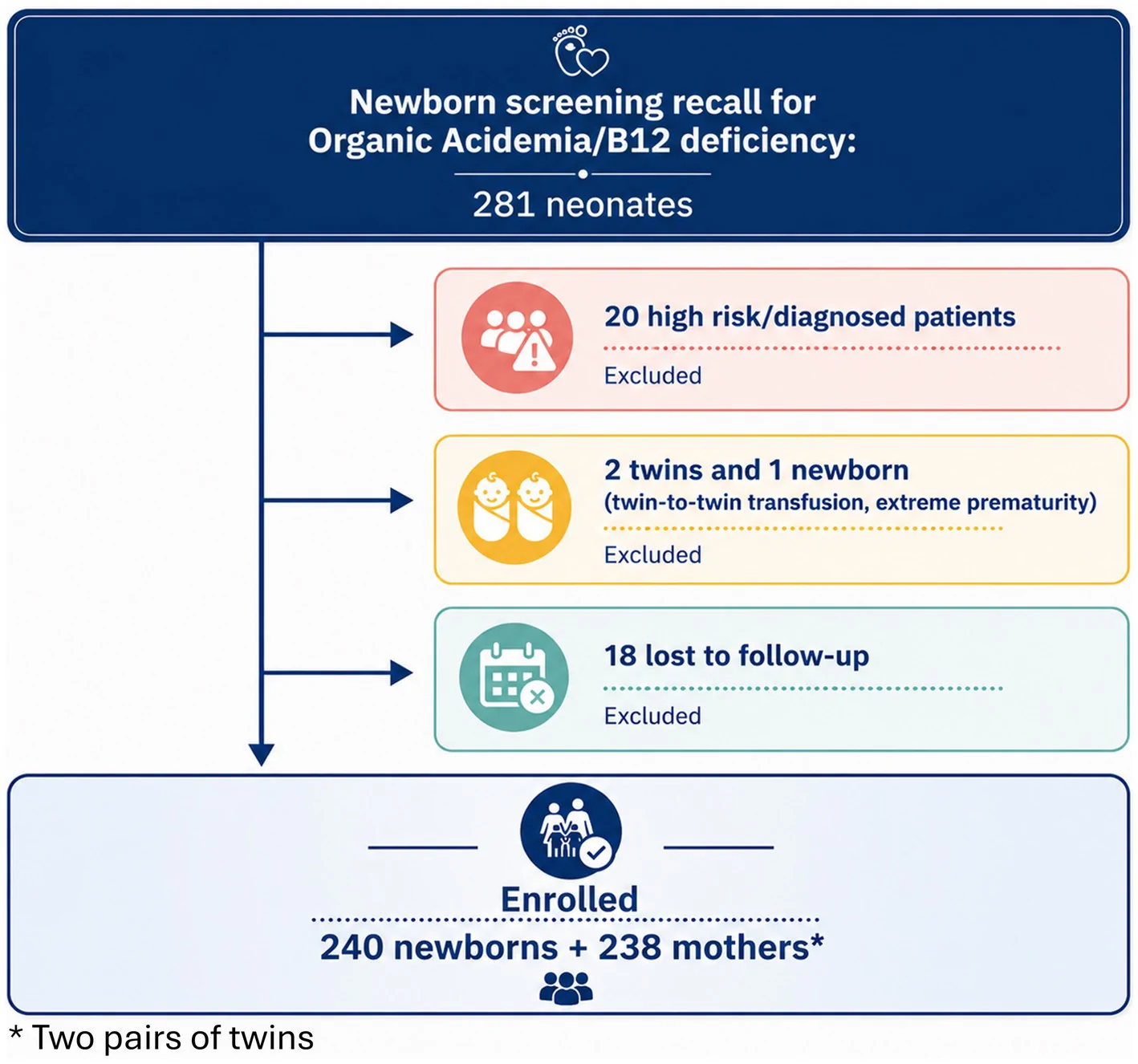
Derivation of the study cohort from newborn screening recalls for suspected vitamin B12 deficiency. This figure was developed with the assistance of ChatGPT 5.5 through an iterative process which is described in Supplementary File 1.

Figures 1, 2 were created from author-generated drafts and graphically refined using ChatGPT (OpenAI). Details of the image generation and refinement process are reported in the Supplementary materials.

## 3 Results

### 3.1 Sample characteristics

Overall, among 281 newborns screened for organic acidaemia/B12 deficiency. Twenty newborns were diagnosed with Organic Acidaemias (12 CblC, 3 MUT0, 1 Mut-, 1 Cbl D, 1 Cbl A, 2 Propionic Acidaemia) and for this reason they were excluded from our retrospective analysis. In addition, 3 patients (2 twins and 1 newborn) with severe prematurity were excluded and 18 patients were lost to follow up. Overall, 240 patients were included in the final sample, all of them received a diagnosis of Vitamin B12 deficiency, and their respective 238 mothers [two couples of twins (Figure 2)].

Within the cohort of enrolled patients, 21 underwent molecular analysis which resulted negative in all patients. Three patients were found carrier of heterozygosis for genes involved in cobalamin metabolism: one for a variant of undefined significance (VUS) in the cubilin gene (*CUBN*); one for a VUS in the methylmalonyl-CoA mutase gene (*MMUT*); one for a pathogenic variant in the *MMACHC* gene.

Infants and mothers’ characteristics are presented in Table 1. The majority of newborns was born from eutocic delivery (72.9%), had a birth weight adequate for gestational age (AGA) (72.5%). Overall, 86.3% was full term and approximately 71.6% was exclusively breastfed at baseline. Table 1 presents C3, Met and MMA values obtained from NBS tests. Mothers were predominately of Caucasian (45.8%) and Indian origin (45.8%). As evidenced, nearly 42.6% of women declared to follow a VEG dietary pattern during pregnancy. About 40 % of mothers underwent investigations to exclude causes of B12 malabsorption, specifically 43% resulted test positive (29.2% for Chronic Atrophic Gastritis; 4.2% Celiac Disease; 7.3% *H. pylori* infection; 1.0% Parasitosis; 1.0% Chronic Atrophic Gastritis plus *H. pylori* infection; 1.0% Chronic Atrophic Gastritis plus parasitosis).

**Table 1:** General characteristics of the expanded newborn screening (ENS) infants and mothers.

Characteristics	Value
Infants	240
Sex	
Males	106 (44.2%)
Females	134 (55.8%)
Parental consanguinity	
First cousins	7 (2.9%)
Twinning	
Twins	12 (5.0%)
No twins	228 (95.0%)
Type of delivery	
Vaginal delivery	175 (72.9%)
Cesarean section	59 (24.6%)
Vacuum-assisted delivery	6 (2.5%)
Birth weight (g)	3,163 [IQR 2750–3,553]
Birth weight category	
Low birth weight (<2,500 g)	33 (13.8%)
Normal birth weight (2500–4,000 g)	194 (80.8%)
High birth weight (>4,000 g)	13 (5.4%)
Birth weight percentiles category	
Small for gestational age [SGA]	38 (15.8%)
Adequate for gestational age [AGA]	174 (72.5%)
Large for gestational age [LGA]	28 (11.7%)
Gestational age (weeks)	39 [38–40]
Gestational age category	
Preterm (<37 weeks)	33 (13.8%)
Term/post-term (≥37 weeks)	207 (86.3%)
NBS recall values (μM)	
Propionylcarnitine (C3, nv < 4.5 μM) (*n* = 221)	5.49 [3.5–6.6]
Methylmalonic Acid (MMA, nv < 1.1 μM) (*n* = 205)	3.4 [1.7–6.4]
Methionine (Met, nv > 8.34 μM) (*n* = 200)	19.0 [11.9–23.9]
Milk-based feeding (*n* = 225)	
Exclusively breastfed	161 (71.6%)
Formula fed	64 (28.4%)
Mothers	238
Age at delivery (years) (*n* = 163)	31 [27–35]
Ethnicity	
Caucasian	109 (45.8%)
Indian	109 (45.8%)
Hispanic or Latino	15 (6.3%)
African	5 (2.1%)
Diet during pregnancy (*n* = 236)	
Omnivorous	136 (57.6%)
Vegetarian	100 (42.4%)
Vitamin B12 malabsorption conditions^a^ (*n* = 96)	
Chronic atrophic gastritis	28 (29.2%)
Celiac disease	4 (4.2%)
*Helicobacter pylori* infection	7 (7.3%)
Parasitosis	1 (1.0%)
Chronic atrophic gastritis + *Helicobacter pylori*	1 (1.0%)
Chronic atrophic gastritis + parasitosis	1 (1.0%)
Negative	54 (56.3%)

Unless otherwise specified, data were available for all patients.

a Excluded from T1 data analysis.

### 3.2 Biochemical parameters at baseline

Table 2 presents plasma and urine variables in the mother–infant dyads collected at each timepoint T0, T1 and T2, as compared to laboratory and NICE reference values. At T0, 56.2% of the newborns showed a mean plasma B12 value below the laboratory reference values (i.e., <191 pg/mL) meanwhile 39.8% showed adequate level (i.e., 191–663 pg/mL). When considering the NICE criteria, classification differs considerably, as 50.0% were classified as “confirmed deficiency,” 35.4% as “probable deficient” and 14.6 with “No deficiency.”

**Table 2:** Plasma and urine variables in infants and mother collected at baseline (T0) and follow-up (T1 and T2), reported as median [IQR] of the values measured, as well as number and percentage (%) of the subjects *versus* the reference range/cut-off values of normality/adequacy.

Biomarker	Reference range/cut-off	Measured concentration		
		**T0**	**T1**	**T2**
Infants				
Plasma Vitamin B12, *N*		226	205	167
Median [IQR]		179.5 [109–271]	1864 [1312–2000]	871 [628–1,177]
Lab reference—deficiency	<191 pg/mL	127 (56.2%)	0 (0.0%)	1 (0.6%)
Lab reference—normal	191–663 pg/mL	90 (39.8%)	12 (5.9%)	45 (26.9%)
Lab reference—above	>663 pg/mL	9 (4.0%)	193 (94.1%)	121 (72.5%)
NICE: confirmed deficiency	<180 pg/mL	113 (50.0%)	0 (0.0%)	1 (0.6%)
NICE: probable deficiency	180–350 pg/mL	80 (35.4%)	2 (1.0%)	8 (4.8%)
NICE: no deficiency	>350 pg/mL	33 (14.6%)	206 (99.0%)	158 (94.6%)
Plasma MMA, *N*		165	202	159
Median [IQR]	<4.0 μmol/L	3.3 [1.6–9.3]	0.6 [0.3–0.8]	0.5 [0.2–0.8]
Higher		74 (44.8%)	2 (1.0%)	1 (0.6%)
Urine MMA, *N*		184	204	162
Median [IQR]	<2 mM/M Creat.ur	51.0 [20.0–131.5]	3.7 [2.8–5.0]	3.0 [2.1–4.3]
Higher		181 (98.4%)	182 (89.2%)	129 (79.6%)
Plasma homocysteine, *N*		199	194	155
Median [IQR]	<15 μmol/L	15.8 [10.0–21.6]	4.9 [4.2–5.6]	5.0 [4.3–6.1]
Higher		105 (52.8%)	0 (0.0%)	0 (0.0%)
Mean cell volume, *N*		141	NA	NA
Median [IQR]	74–108 fl^*^	94.0 [89.5–98.6]		
Lower		0 (0.0%)		
Within		138 (97.9%)		
Higher		3 (2.1%)		
Mothers				
Plasma vitamin B12, *N*		191	35	NA
Median [IQR]		216 [142.2–306]	381 [285–471]	
Lab reference—deficiency	<191 pg/mL	83 (43.5%)	2 (5.7%)	
Lab reference—normal	191–663 pg/ml	106 (55.5%)	29 (82.9%)	
Lab reference—above	>663 pg/mL	2 (1.0%)	4 (11.4%)	
NICE: confirmed deficiency	<180 pg/mL	76 (39.8%)	2 (5.7%)	
NICE: probable deficiency	180–350 pg/mL	81 (42.4%)	15 (42.9%)	
NICE: no deficiency	>350 pg/mL	34 (17.8%)	18 (51.4%)	
Plasma folate, *N*		128	28	NA
Median [IQR]	4.6–18.7 ng/mL	8.3 [4.7–14.3]	5.3 [4.2–9.8]	
Lower		30 (23.4%)	10 (35.7%)	
Within		81 (63.3%)	15 (53.6%)	
Higher		17 (13.3%)	3 (10.7%)	
Plasma homocysteine, *N*		157	24	NA
Median [IQR]	<15 μmol/L	12.8 [9.4–17.6]	8.7 [7.4–11.3]	
Higher		56 (35.7%)	2 (8.3%)	
Haemoglobin, *N*		115	26	NA
Median [IQR]	>12 g/dL	12.7 [11.8–13.6]	13.0 [12.1–13.3]	
Lower		41 (35.7%)	5 (19.2%)	
Hematocrit, *N*		106	25	NA
Median [IQR]	35.0–47.0%	39.1 [36.3–41.0]	38.4 [37.1–40.6]	
Lower		15 (14.2%)	1 (4.0%)	
Within		91 (85.8%)	24 (96.0%)	
Higher		0 (0.0%)	0 (0.0%)	
Mean cell volume, *N*		114	24	NA
Median [IQR]	82.0–97.0 fl	88.0 [83.8–92.4]	86.8 [81.0–87.9]	
Lower		24 (20.2%)	7 (29.2%)	
Within		75 (65.8%)	16 (66.7%)	
Higher		16 (14.0%)	1 (4.2%)	

*Median values in children 3-6 months.

Regarding plasma Hcy and MMA in the newborns, concentrations were above the laboratory reference values in 52.8 and 44.8%, respectively. Urinary MMA exceeded the reference cutoff (<2 mmol/mol creatinine) in 98.4% of newborns, with a median concentration of 51.0 mmol/mol creatinine [20.0–131.5], approximately 25-fold higher than the upper reference limit. MCV values were within the normal range in most newborns; however, macrocytosis was observed in 2.1% of cases.

When B12 status among mothers was considered (Table 2), 43.5% were below the laboratory reference value, whilst almost half of women (about 55.5%) were within the range of normality. When using NICE cutoff, a confirmed and probable deficiency were observed in 39.8 and 42.4%, respectively, with only 17.8% of mothers having normal values. Regarding the other biomarkers, nearly one third of women had mild hyperhomocysteinemia (35.7%), meanwhile 23.4% of mothers were deficient in folate. About one third of mothers had a condition of anaemia (<12 g/dL) at baseline (35.7%), but only 14% of all mothers presented with macrocytosis.

In newborns an inverse correlation was found between plasma B12 and plasma MMA (*ρ* = −0.29; *p* = 0.0002), urine MMA (*ρ* = −0.22; *p* = 0.0003), Hcy (*ρ* = −0.52; *p* < 0.0001), and MCV (*ρ* = −0.25; *p* = 0.0027) (results not shown). In mothers an inverse correlation was found between plasma B12 and both Hcy (*ρ* = −0.50; *p* < 0.0001) and MCV (*ρ* = −0.23; *p* = 0.014).

### 3.3 Correlations in mother–newborn dyads at T0

**Figure 3:**
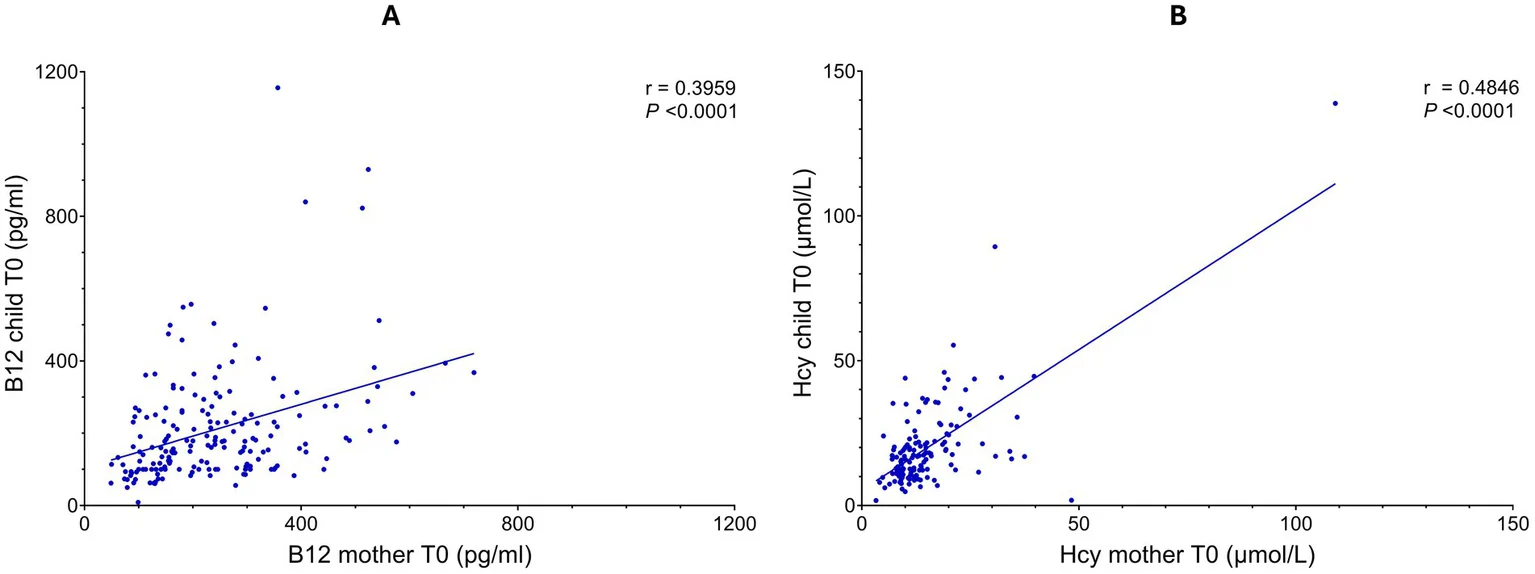
Association between maternal and child levels of vitamin B12 **(A)** and homocysteine **(B)**. HCY, homocysteine; T0, baseline.

**Figure 4:**
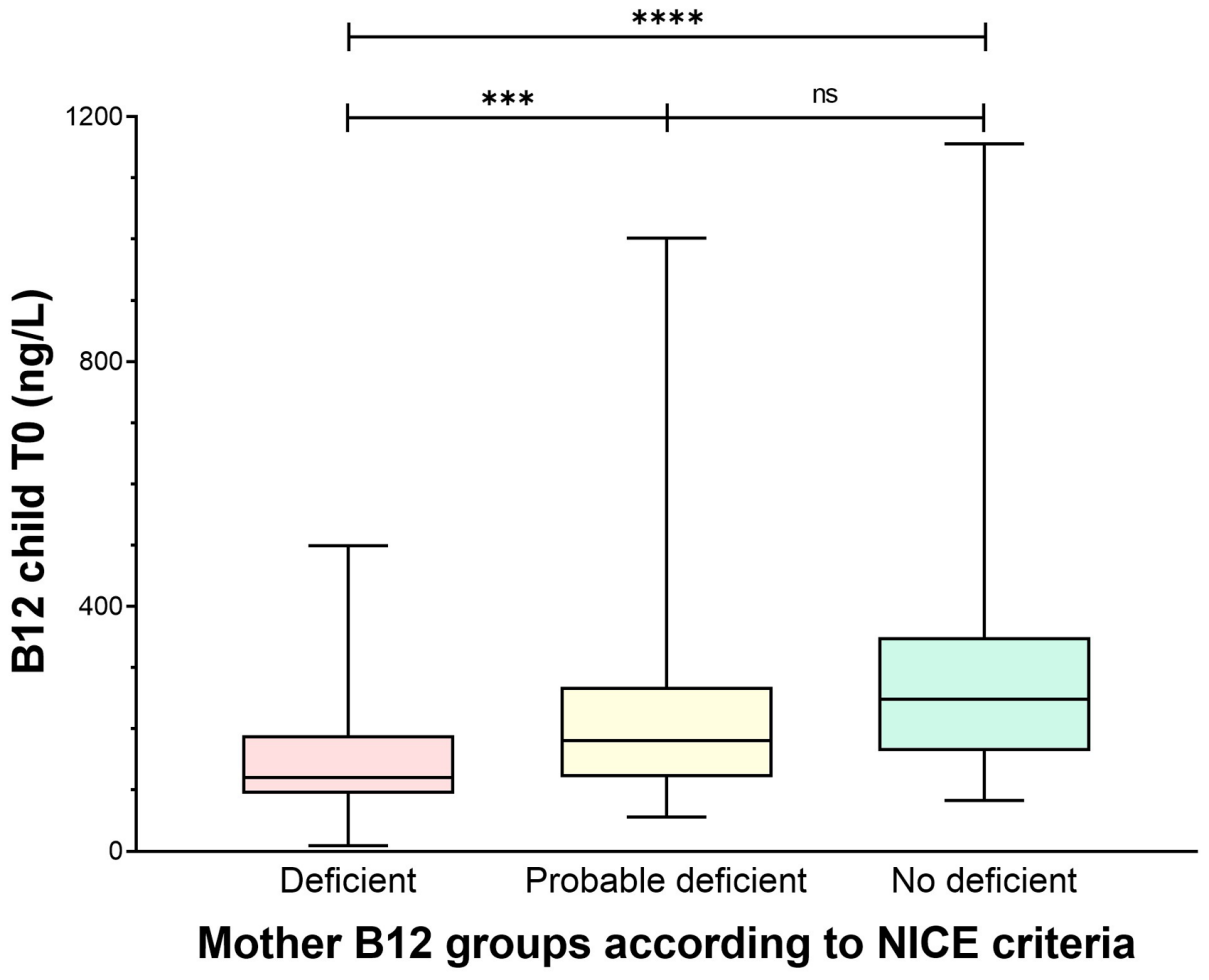
Child B12 values at T0 according to maternal vitamin B12 status classified using the NICE criteria.

Positive correlations in vitamin-B12 (*p* < 0.01) and Hcy (*p* < 0.01) were evidenced in the mother–newborn dyads’ concentrations (Figures 3A,B, respectively). A significant difference was found in B12 levels among infants born to mothers with B12 deficient, B12 probable deficient and no deficient (*p*_for trend_<0.0001) (Figure 4). Specifically, infants born to mothers classified “Deficient” showed a median B12 level of 120.0 pg/mL that was has significantly lower compared to both “Probable deficient” (181.0 pg/mL, *p* = 0.0002) and “No Deficiency” (249.0 pg/mL, *p* < 0.0001); while infants born from mother “Probable deficient” were not significantly different from “No Deficiency” mothers (*p* = 0.22).

Analysing only newborn with a pathological Hcy value (≥15 μmoL/L) (52.5%), NICE criteria identified significantly more patients as vitamin B12 deficient/at risk than laboratory reference ranges (93.1% vs. 74.5%) according to McNemar test (*p* < 0.0001). When considering infants with pathological MMA value (≥4.0 μmol/L) (40.9%), NICE criteria identified significantly more patients as vitamin B12 deficient/at risk than laboratory reference ranges (88.4% vs. 67.4%) according to McNemar test (*p* < 0.0039).

### 3.4 Dietary factors and B12 biomarkers

When considering the dietary pattern, plasma B12 concentration in OMs mothers was higher than in VEG [242.5 pg/mL (152–347.8) vs. 171 pg/mL (124–246), respectively; *p* = 0.0006], while Hcy levels were not significantly different [11.8 μmoL/L (8.8–18.1) vs. 14.0 μmoL/L (10.5–17.5), respectively; *p* = 0.054]. Meanwhile, plasma B12 and plasma MMA concentration in newborns from OMs mothers were not significantly different from newborns from VEG mothers [B12 180.5 pg/mL (113.3–279.0) vs. 162.5 pg/mL (108.5–263.0), *p* = 0.39; MMA 3.8 μmoL/L (1.46–10.1) vs. 2.9 μmoL/L (1.69–7.8); *p* = 0.52, respectively].

Plasma B12 was significantly lower in exclusively breastfed infants compared to formula fed [161.0 pg/mL (104.0–236.0) vs. 226 pg/mL (135.0–382.0), *p* = 0.0008]. Interestingly, plasma MMA and urinary MMA were significantly higher in formula fed infants compared to breastfed ones [plasma MMA 5.2 μmoL/L (1.8–11.5) vs. 3.0 μmoL/L (1.4–7.6) *p* = 0.049; urinary MMA 70.0 mmol/mol creatinine (28.0–160.0) vs. 42.4 mmol/mol creatinine (15.0–117.0), *p* = 0.02]. No significant difference were observed for Hcy and MCV according to feeding type.

### 3.5 Longitudinal changes in biochemical parameters at T1 and T2

**Figure 5:**
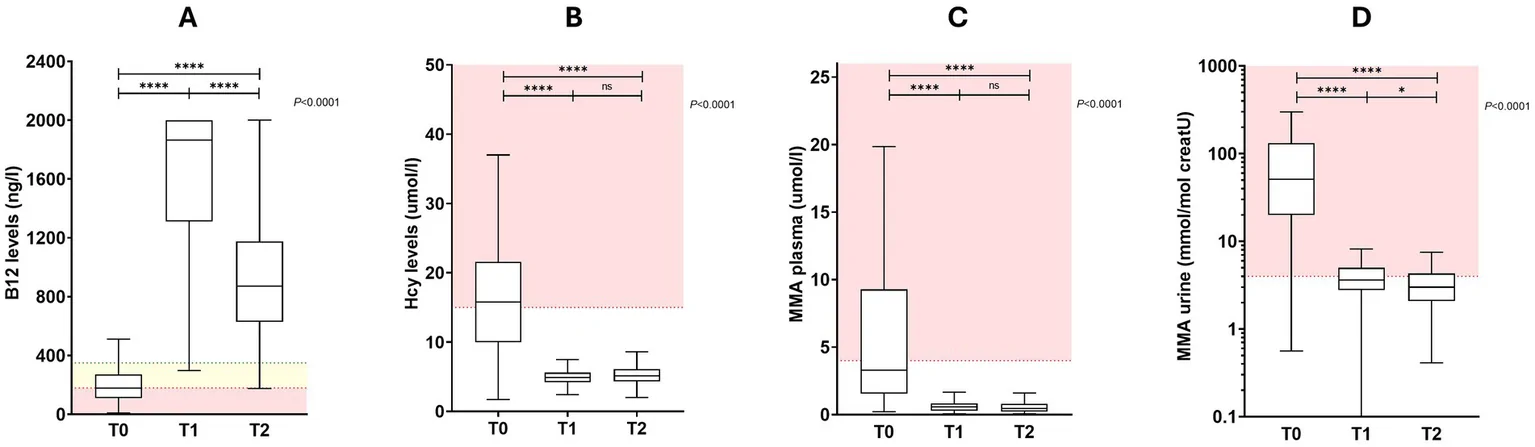
Longitudinal changes in vitamin B12 status biomarkers during follow-up. Box-and-whisker plots show serum vitamin B12 **(A)**, plasma total homocysteine **(B)**, plasma methylmalonic acid **(C)**, and urinary methylmalonic acid **(D)** at baseline (T0), during vitamin B12 supplementation (T1), and after discontinuation of supplementation following complementary feeding (T2). Panel **(A)** yellow and red color as for NICE criteria, panels **(B–D)** red colour for pathological value according to lab.

After 2-months of B12 supplementation (T1) and after 2-month of CF initiation and B12 supplementation discontinuation (T2), a marked improvement in infant vitamin B12 status was observed over time. Median plasma vitamin B12 concentrations increased from 179.5 pg/mL [109.8–271] at baseline to 1864 pg/mL [1312.0–2000.0] at T1. Following supplementation discontinuation, vitamin B12 concentrations significantly declined to 871 pg/mL [628–1,177] at T2, while remaining within the normal range in the vast majority of infants (all pairwise comparisons *p* < 0.0001) (Figure 5A). According to laboratory reference ranges, the prevalence of vitamin B12 deficiency decreased from 56.2% at T0 to 0% at T1 and 0.6% at T2. Similarly, using NICE criteria, confirmed deficiency decreased from 50.0% at baseline to 0% at T1 and 0.6% at T2, whereas probable deficiency declined from 35.4 to 1.0 and 4.8%, respectively. Consequently, 99.0% of infants at T1 and 94.6% at T2 were classified as having no vitamin B12 deficiency according to NICE criteria (Table 2). Parallel improvements were observed in functional biomarkers of vitamin B12 status. Hcy, plasma MMA, and urinary MMA concentrations decreased markedly from T0 to T1 and remained significantly lower than baseline at T2 (all comparisons vs. T0, *p* < 0.0001), with median values falling below their respective reference cut-offs (Figures 5B–D, respectively). No significant differences were observed between T1 and T2 for Hcy and plasma MMA, indicating sustained biochemical normalization after supplementation discontinuation. In contrast, urinary MMA showed a further modest decrease between T1 and T2 (*p* = 0.043). Notably, the proportion of infants with elevated plasma MMA and Hcy almost completely normalized during follow-up, decreasing from 44.8 to 0.6% and from 52.8 to 0%, respectively. In contrast, urinary MMA remained above the reference cut-off in a substantial proportion of infants, despite a progressive decline from 98.4% at baseline to 89.2% at T1 and 79.6% at T2 (Table 2).

Regarding maternal data (Table 2), at T1 the percentage of women falling below the lowest plasma B12 level of the commonly used reference interval decreased from 43.5 to 5.7%. Nevertheless, plasma B12 inadequacy remained evident when applying the NICE criteria, with 5.7% of women classified as having confirmed deficiency and 42.9% as having probable deficiency at T1. Median vitamin B12 concentration increased from 216 pg/mL at T0 to 381 pg/mL at T1. In contrast, supplementation improved Hcy status as the percentage of women above the highest level of the reference interval decreased from about 35.7–8.3%.

## 4 Discussion

We presented data from 240 NBS-newborns pooled at our RCC, screened positive for the MMA/B12 Deficiency risk, with a final diagnosis of secondary vitamin-B12 deficiency, and not an Organic Acidemias. At recall, most of the newborns did not meet vitamin-B12 sufficiency values while showing 25-fold increase compared to the normal values of urine MMA. As expected, even though serum folate was sufficient, B12 deficiency was common among mothers, in accordance with literature acknowledging that worldwide, low B12 status has a frequency of 10–50% among women of childbearing-age and pregnant (7).

Vitamin B12 deficiency is one of the most common vitamin deficiencies; however, in Italy there are currently no national guidelines defining the most appropriate biomarkers for its diagnosis. Serum B12 is frequently used, although it is considered less reliable because a large proportion of circulating cobalamin is bound to haptocorrin and is not bioavailable to cells (19). In contrast, holotranscobalamin reflects the biologically active fraction of vitamin B12, but diagnostic cut-off values remain controversial. From March 2024 the NICE recommends to use either total B12 or active B12 (holotranscobalamin) for individuals aged 16 years and older, with active B12 as the first-line test during pregnancy (3). Functional markers such as Hcy and MMA have been proposed to fill this diagnostic gap, but their levels can be affected by other factors, making them less reliable as stand-alone indicators (20). This lack of consensus complicates not only clinical practice but also research comparability. Moreover, the lack of harmonization across available assays significantly complicates the interpretation of results (21, 22). HoloTC provides a more direct insight into the usable B12 pool, and this is particularly relevant in pregnancy, where total B12 may appear falsely low due to haemodilution or reduced levels of haptocorrin, the protein that binds most circulating B12 (23). Similarly, newborns can show misleading values because human milk is rich in unsaturated haptocorrin, which affects the interpretation of total B12 levels (24). Both underestimation and overestimation of B12 status can lead to clinical risk.

A gestational prolonged state of B12 deficiency may impact critically on pregnancy outcomes (i.e., low birth weight risk, preterm birth etc.) and foetal neurological/psychomotor development (25). In line with other findings, our analysis evidenced that infant B12 status depended on the pregnant mother’s one, as demonstrated by the significant correlations assessed in the vitamin status biomarkers between mothers and babies at recall in the very first days of life (18, 26, 27). Specifically, it emerged that maternal Hcy levels were the most strongly correlated to the neonatal B12 status, confirming that maternal plasma Hcy dosage in pregnancy could be a good diagnostic tool to endow the mother with early tailored B12-supplementation, thus avoiding a deficit in the child. These findings support a mother-centred approach to prevention, whereby identification of maternal vitamin B12 deficiency during pregnancy may allow timely nutritional counselling and supplementation before neonatal biochemical abnormalities become clinically relevant. From a clinical perspective, maternal assessment may therefore represent an effective strategy to reduce the burden of neonatal vitamin B12 deficiency rather than relying exclusively on postnatal identification through newborn screening. Nevertheless, the potential benefits of antenatal supplementation should be considered alongside the possibility that maternal treatment may partially normalize neonatal biochemical markers, potentially reducing the detectability of mild or borderline B12-responsive metabolic disorders at newborn screening. However, given the retrospective design, these correlations should be interpreted as associative and cannot define causality or establish universal screening thresholds.

Indeed, despite Hcy concentrations are relatively unaffected by mild B12 deficiency, they rise rapidly when plasma vitamin-B12 falls below 300 pmol/L (equals to 406 pg/mL) (2).

Our findings further support the concept that conventional laboratory reference ranges may underestimate the burden of vitamin B12 deficiency in mother–infant dyads. While 39.8% of newborns had plasma vitamin B12 concentrations within the laboratory normal range (191–663 pg/mL), application of the NICE criteria reclassified the majority of these infants as confirmed deficiency (50.0%) and probable deficiency (35.4%), leaving only 14.6% categorized as having no deficiency. This observation suggests that laboratory reference intervals may be poorly sensitive in identifying newborns at risk of functional vitamin B12 deficiency.

The clinical relevance of this reclassification is reinforced by the clear relationship between maternal and neonatal vitamin B12 status. Infants born to mothers classified as vitamin B12 deficient according to NICE criteria showed significantly lower vitamin B12 concentrations than those born to mothers with probable deficiency or no deficiency, supporting the biological plausibility of the NICE stratification and its ability to capture varying degrees of risk within the mother–infant dyad. Notably, neonatal vitamin B12 concentrations did not differ significantly between infants born to mothers classified as having probable deficiency and those born to mothers without deficiency. This finding should not be interpreted as evidence that maternal vitamin B12 insufficiency is clinically irrelevant, since neonatal stores may initially compensate for marginal maternal status and functional consequences could emerge later in infancy.

More recently, a recent study in adults with macrocytic anaemia proposed optimized diagnostic thresholds for total vitamin B12, suggesting that concentrations ≤257 ng/L indicate severe deficiency, while levels between 258 and 341 ng/L may reflect early depletion and require confirmation with homocysteine, supporting the need for harmonized diagnostic cut-offs (28).

The role of dietary habits cannot be disregarded when considering maternal B12 status. In our cohort, nearly half of the mothers were of Indian origin and 42.6% reported following a VEG dietary pattern during pregnancy. In line with previous studies, maternal dietary habits were associated with significant differences in biochemical status (14), in fact, in our cohort VEG mothers showed lower plasma B12 concentrations than OMs mothers. There is evidence that a functional B12 deficiency is common in vegetarians (29, 30). Moreover, in a large retrospective Canadian cohort-study, South Asian ethnicity appeared to be a significantly negative predictor of B12 status during pregnancy (31). Meat exclusion is culturally rooted in some ethnicity, and it is increasingly popular also in the Western word (32). Hence, any physiological phase with an increased vitamin requirement, such as pregnancy, lactation and infancy may represent a further risk for B12-deficiency (2, 29, 30). According to Italian Dietary Reference Values (SINU, LARN 2024) (33), during pregnancy and lactation the dietary B12 adequate intake recommended increases up to 4.5 μg/day and 5 μg/day, respectively, as the woman tends to keep reserves whilst transmitting the circulating vitamin-B12 to child. Despite the differences in maternal vitamin B12 status according to dietary pattern adoption, neonatal plasma B12 and MMA concentrations did not differ significantly according to maternal diet, suggesting that additional determinants may influence neonatal vitamin B12 metabolism. Furthermore, the absence of significant differences in neonatal vitamin B12 status should be interpreted with caution, as our broad classification of maternal dietary patterns may have masked differences among vegetarian subtypes with markedly different vitamin B12 intake and supplementation practices.

Indeed, as expected, feeding type was more strongly associated with neonatal vitamin B12 status. Exclusively breastfed infants showed significantly lower plasma B12 concentrations than formula-fed infants, supporting previous evidence that infant vitamin B12 status is closely dependent on maternal stores and breastmilk vitamin B12 content (6, 34, 35). The persistence of elevated plasma and urinary MMA in formula-fed infants, despite higher plasma B12 concentrations, supports the hypothesis that many infants had experienced vitamin insufficiency before formula introduction, with circulating B12 recovering more rapidly than functional metabolic abnormalities.

Regarding the possibility that the low B12 status observed in our NBS population was related to systemic malabsorption disorders in the infants, their contribution appears limited. Conversely, vitamin B12 malabsorption in mothers may have played a more relevant role than expected. Among the approximately 40% of mothers who underwent investigations, 43% were diagnosed with an underlying condition potentially affecting vitamin B12 absorption, most commonly chronic atrophic gastritis, followed by *H. pylori* infection and celiac disease. However, these findings should be interpreted cautiously, as malabsorption disorders were not systematically investigated in all mothers.

A novel aspect of the present study is the longitudinal assessment of vitamin B12 biomarkers up to 8 months of age. As expected, both 2 months of vitamin B12 supplementation and the subsequent introduction of complementary feeding were associated with a marked improvement in overall vitamin status. Plasma vitamin B12 concentrations increased substantially, accompanied by a pronounced reduction in Hcy and plasma MMA levels. Following supplementation discontinuation, plasma vitamin B12 concentrations decreased; however, values remained within the normal range in the vast majority of infants, suggesting maintenance of an adequate vitamin B12 status after the introduction of complementary foods. Notably, urinary MMA remained elevated in a large proportion of infants despite normalization of the other biochemical markers of vitamin B12 status, highlighting the different behavior of this biomarker during follow-up. The persistence of urinary MMA above the reference cutoff in a substantial proportion of infants, despite normalization of plasma vitamin B12, homocysteine, and plasma MMA, may reflect the higher sensitivity and different kinetics of urinary MMA compared with circulating biomarkers. Urinary MMA may remain mildly elevated after correction of vitamin B12 status because of residual intracellular metabolic adaptation, renal handling, hydration status, creatinine correction, and age-related variability. In addition, currently used urinary MMA reference cutoffs may not be fully optimized for infants followed after newborn screening recall. In our cohort, however, urinary MMA showed a marked quantitative decrease over time, suggesting biochemical improvement despite persistent values above the laboratory threshold.

In contrast, the available follow up data in mothers suggested a less pronounced biochemical response after supplementation. However, maternal follow-up was available for only a limited subset of women, substantially reducing the statistical power and limiting the interpretation and generalizability. Within this subgroup, median plasma vitamin B12 concentrations increased from 216 to 381 pg/mL after supplementation, although a substantial proportion of women remained within the deficient or probable deficiency categories. This finding may partly reflect suboptimal adherence to supplementation. In addition, the high proportion of women of foreign origin in our cohort may have represented a barrier to effective counselling and follow-up.

### 4.1 Strengths and limitations

Among the strengths of our analysis, this is one of the few studies addressing the issue of the current Vitamin-B12 status in the mother–infant dyads in Italy. Moreover, our findings likely contribute to the up-to-date discussion about B12 biology, including available and potential biomarkers of status (2, 4, 36). Our results highlight it should be useful to re-evaluate the reference values for B12 given that those commonly used could lead to misplace an important segment of subjects with latent/subclinical deficit, which may induce biochemical alterations. Indeed, no consensus exists about which cut-off values should be applied and which is the best marker or markers’ combination to assess vitamin-B12 status (2, 15), which hampers interpretation difficult for general practitioners. For example, our results evidenced an absence of clear haematological changes in the mother–newborn pairs with vitamin-B12 values compatible with deficiency status, even in cases with more serious deficiencies (<191 pg/mL), which can be explained by the need of a fairly long time for a deficiency to get manifested, therefore implying it is not possible to use the increased MCV as a parameter of suspected B12 deficiency.

Importantly, although our findings highlight the usefulness of the newborn screening pathway in identifying secondary neonatal vitamin B12 deficiency, the present cohort represents a selected population of infants recalled after positive newborn screening, and not a population-based sample of all mother–infant dyads. Therefore, the frequency of biochemical abnormalities observed in this study cannot be used to estimate the prevalence of vitamin B12 deficiency in the general neonatal population. The results should instead be interpreted as describing the biochemical and clinical characteristics of dyads identified through the newborn screening pathway.

This study has some limitations that can affect the interpretation and generalizability of results. First, its retrospective design limited the completeness of data collection and precluded any inference of causal relationships. Second, the availability of biochemical data varied across biomarkers and time points (T0, T1, and T2) owing to missed follow-up visits, refusal of blood sampling, and difficulties in urine or venous sample collection in infants. Furthermore, maternal follow-up data could not be collected systematically, as adult women were routinely referred to community healthcare services after the initial assessment and subsequent laboratory investigations were often performed outside our center. The study did not include standardized growth or neurodevelopmental outcome measures; therefore, the clinical significance of the biochemical abnormalities observed at baseline and during follow-up, including persistent mild urinary MMA elevation, could not be directly assessed. The broad classification of maternal dietary patterns during pregnancy, based on information routinely recorded in clinical charts, limited differentiation among vegetarian subtypes (e.g., pescatarian, lacto-ovo vegetarian, and vegan diets), which may differ substantially in vitamin B12 intake and supplementation practices. Finally, the lack of a control group of mother–infant dyads not identified through NBS limits the possibility of performing more comprehensive clinical, dietary, and biochemical comparisons.

## 5 Conclusion

Taken together, data from this selected newborn-screening-recalled population support the relevance of maternal vitamin B12 status for neonatal biochemical status and highlight the role of expanded newborn screening in identifying secondary neonatal vitamin B12 deficiency. However, because of the retrospective design and the absence of a mother–infant control group not identified through newborn screening, these findings should not be generalized to the overall pregnant or neonatal population, nor should they be interpreted as sufficient evidence to define universal screening recommendations. Rather, they support the need for greater awareness of maternal risk factors for vitamin B12 deficiency, including poorly planned vegetarian/vegan diets and subclinical malabsorption, particularly during pregnancy and lactation. Further adequately designed prospective studies including in-depth assessments of maternal dietary habits, dietary patterns, supplementation, malabsorption conditions, and clinical outcomes are warranted. Implementing a more detailed maternal dietary assessment in routine clinical practice may also improve the identification of pregnancies at increased risk of vitamin B12 deficiency. Greater awareness among gynaecologists, paediatricians, and family doctors may help create a prevention network aimed at early identification of women most at risk of B12 insufficiency, allowing timely supplementation and prevention of neonatal deficiency. At the same time, further studies are needed to clarify whether maternal vitamin B12 supplementation during pregnancy may influence newborn screening biomarkers and thereby affect the identification of neonatal vitamin B12 deficiency.

## Data Availability

The raw data supporting the conclusions of this article will be made available by the authors, without undue reservation.
