# Supplementary material for: Vitamin-B12 status among the mother–infant dyad: an explorative overview in a single centre in Lombardy
Source: Front Nutr. 2026 Sep 11;13:1903144. doi: 10.3389/fnut.2026.1903144 (PMC13614015; doi:10.3389/fnut.2026.1903144)
Supplement: Supplementary file 1 [file Supplementary_file_1.DOCX]

**Supplementary Materials 1**

The study timeline figure (**Figure 1**) was developed through an iterative human–AI collaborative process using ChatGPT (OpenAI). The authors provided the following scheme and then refine data regarding study design, clinical workflow, timing of assessments, supplementation schedule, and all textual content.

The AI-assisted process included:

- **Graphical synthesis**: Transformation of a text-based study protocol into a publication-ready timeline infographic with standardized iconography, color coding, and visual hierarchy.
- **Workflow visualization**: Representation of the newborn screening recall pathway, follow-up visits, complementary feeding milestones, and vitamin B12 supplementation schedule within a unified chronological framework.
- **Iterative refinement**: Multiple rounds of prompt-guided modifications were performed to optimize layout, positioning of study milestones, timeline structure, icon selection, and graphical representation of treatment phases according to the authors' specifications.

The authors retained full control over the scientific content and performed a comprehensive manual review of the final figure to verify the accuracy of all study procedures, timelines, labels, and clinical information before publication.

**Figure 2** was generated by providing the following schematic flowchart to ChatGPT (OpenAI). The AI was utilized to transform this conceptual draft into a high-resolution infographic.

The iterative generative process included:

- **Graphical synthesis:** transformation of the authors’ schematic draft into a publication-ready flowchart with standardized layout, professional iconography, and improved visual hierarchy.
- **Visual optimization:** refinement of proportions, typography, color palette, spacing, and alignment to enhance readability and suitability for scientific publication.
- **Iterative refinement:** implementation of multiple author-directed modifications, including corrections to labels, participant counts, exclusion categories, box dimensions, and graphical elements.

All study data, participant numbers, inclusion and exclusion criteria, and the overall figure structure were defined by the authors. The AI-assisted process was limited to graphical design and layout optimization. The authors subsequently performed a comprehensive manual review to verify the accuracy of all numerical values, labels, and study-specific information presented in the final figure.
